# Overcoming multidrug resistance by knockout of ABCB1 gene using CRISPR/Cas9 system in SW620/Ad300 colorectal cancer cells

**DOI:** 10.1002/mco2.106

**Published:** 2021-12-16

**Authors:** Zi‐Ning Lei, Qiu‐Xu Teng, Zhuo‐Xun Wu, Feng‐Feng Ping, Peng Song, John N.D. Wurpel, Zhe‐Sheng Chen

**Affiliations:** ^1^ Department of Pharmaceutical Sciences College of Pharmacy and Health Sciences St. John's University Queens New York USA; ^2^ Department of Reproductive Medicine Wuxi People's Hospital Affiliated to Nanjing Medical University Wu‐xi Jiangsu P.R. China; ^3^ Key Laboratory of Prevention and Treatment for Chronic Diseases by TCM in Gansu Province Affiliated Hospital of Gansu University of Chinese Medicine Lanzhou P.R. China

**Keywords:** ABCB1, colorectal cancer, CRISPR/Cas9, multicellular tumor spheroids, multidrug resistance

## Abstract

Multidrug resistance (MDR) has been extensively reported in colorectal cancer patients, which remains a major cause of chemotherapy failure. One of the critical mechanisms of MDR in colorectal cancer is the reduced intracellular drug level led by the upregulated expression of the ATP‐binding cassette (ABC) transporters, particularly, ABCB1/P‐gp. In this study, the CRISPR/Cas9 system was utilized to target *ABCB1* in MDR colorectal cancer SW620/Ad300 cell line with ABCB1 overexpression. The results showed that stable knockout of *ABCB1* gene by the CRISPR/Cas9 system was achieved in the MDR cancer cells. Reversal of MDR against ABCB1 chemotherapeutic drugs increased intracellular accumulation of [^3^H]‐paclitaxel accumulation, and decreased drug efflux activity was observed in MDR SW620/Ad300 cells after *ABCB1* gene knockout. Further tests using the 3D multicellular tumor spheroid model suggested that deficiency in ABCB1 restrained tumor spheroid growth and restore sensitivity to paclitaxel in MDR tumor spheroids. Overall, the CRISPR/Cas9 system targeting the *ABCB1* gene can be an effective approach to overcome ABCB1‐mediated MDR in colorectal cancer SW620/Ad300 cells.

## Graphical abstract

In this study, the CRISPR/Cas9 system was utilized to target ABCB1 in multidrug resistant (MDR) colorectal cancer SW620/Ad300 cell line with ABCB1 overexpression. Knockout of ABCB1 gene resulted in reversal of MDR against ABCB1 chemotherapeutic drugs, increased intracellular accumulation of [^3^H]‐paclitaxel accumulation, and decreased drug efflux activity in MDR colon cancer cells. In a 3D multicellular tumor spheroid model, deficiency in ABCB1 restrained tumor spheroid growth and restore sensitivity to paclitaxel in MDR tumor spheroids.

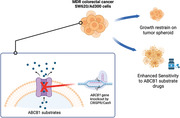

## 1 INTRODUCTION

Chemotherapy, as one of the preferred treatment strategies in combating colorectal cancer, can be adopted at different stages of colorectal cancer, and used as adjuvant therapy before or after surgery.^1^, ^2^, ^3^, ^4^ However, multidrug resistance (MDR) in cancer, which refers to the reduced responsiveness of cancer cells to multiple chemotherapeutic drugs that are not structurally or functionally related,^5^ has been reported extensively in patients with colorectal cancer, leading to impairment in the chemotherapy success.^6^ MDR phenotype in cancer cells can be intrinsic resistance against standard chemotherapy at diagnosis or acquired by prolonged exposure to chemotherapy.^5^, ^7^ Various mechanisms have been discovered to be involved in MDR of colorectal cancer including instability of chromosome,^8^ mutation of drug target,^9^ impaired apoptotic pathways,^10^ and decrease in intracellular drug level due to reduced drug uptake or lifted drug efflux mediated by transporters.^11^ In particular, the upregulated expression of ATP‐binding cassette (ABC) transporters that function as pumps eliminating drugs from cancer cells is a predominant cause of colorectal cancer MDR.^6^, ^11^ The ABC transporter superfamily consists of 49 members categorized into seven subfamilies, which are termed from ABCA to ABCG.^12^ Among the members, ABCB1, also known as P‐glycoprotein (P‐gp), is one of the most common contributors to MDR in colorectal cancer. ABCB1 is a transmembrane transporter that is extensively expressed in various types of tissues, particularly at a high level in the epithelial cells of the colon, small intestine, kidney proximal tubules, bile ducts, and pancreatic ducts.^9^ With a vast spectrum of substrates, ABCB1 plays an important physiological role in protecting tissues by extruding various xenobiotics and toxicants.^13^ On the other hand, tumors expressing ABCB1 gain resistance to many widely used chemotherapeutic drugs that are ABCB1 substrates, such as doxorubicin, paclitaxel, and vincristine,^14^, ^15^ leading to failure of chemotherapy.

To overcome ABCB1‐mediated MDR, three generations of small molecule ABCB1 inhibitors and some natural products considered as the fourth‐generation ABCB1 inhibitors have been developed for coadministration with conventional anticancer drugs thereby increasing intracellular accumulation of anticancer drugs.^16^ Although promising potency in inhibiting ABCB1 function has been reported from cell‐based research and animal studies, none of these compounds achieved approval as ABCB1 inhibitors in clinical application due to undesirable pharmacokinetic profiles or adverse effects.^12^ Another approach to surmount MDR induced by ABCB1 overexpression is to suppress the expression level. Experimental gene therapies using ribozymes, antisense oligonucleotides, as well as small interfering RNAs have been developed and demonstrated success in reversing MDR in cancer cells by downregulating ABCB1 expression.^17^, ^18^, ^19^ In the past decade, the clustered regularly interspaced short palindromic repeats (CRISPR)/CRISPR‐associated 9 (Cas9) gene‐editing system has been developed and utilized for precise gene expression regulation in cell lines and animal models for cancer research.^18^ In the present study, a CRISPR/Cas9 was applied to establish *ABCB1* gene knockout cell models from an ABCB1 overexpressing MDR colorectal cancer cell line SW620/Ad300. The effects of *ABCB1* knockout on drug resistance phenotype, intracellular drug accumulation, and tumor spheroid morphology were investigated.

## 2 RESULTS

### 2.1 Stable knockout of ABCB1 in SW620/Ad300 cells

**FIGURE 1:**
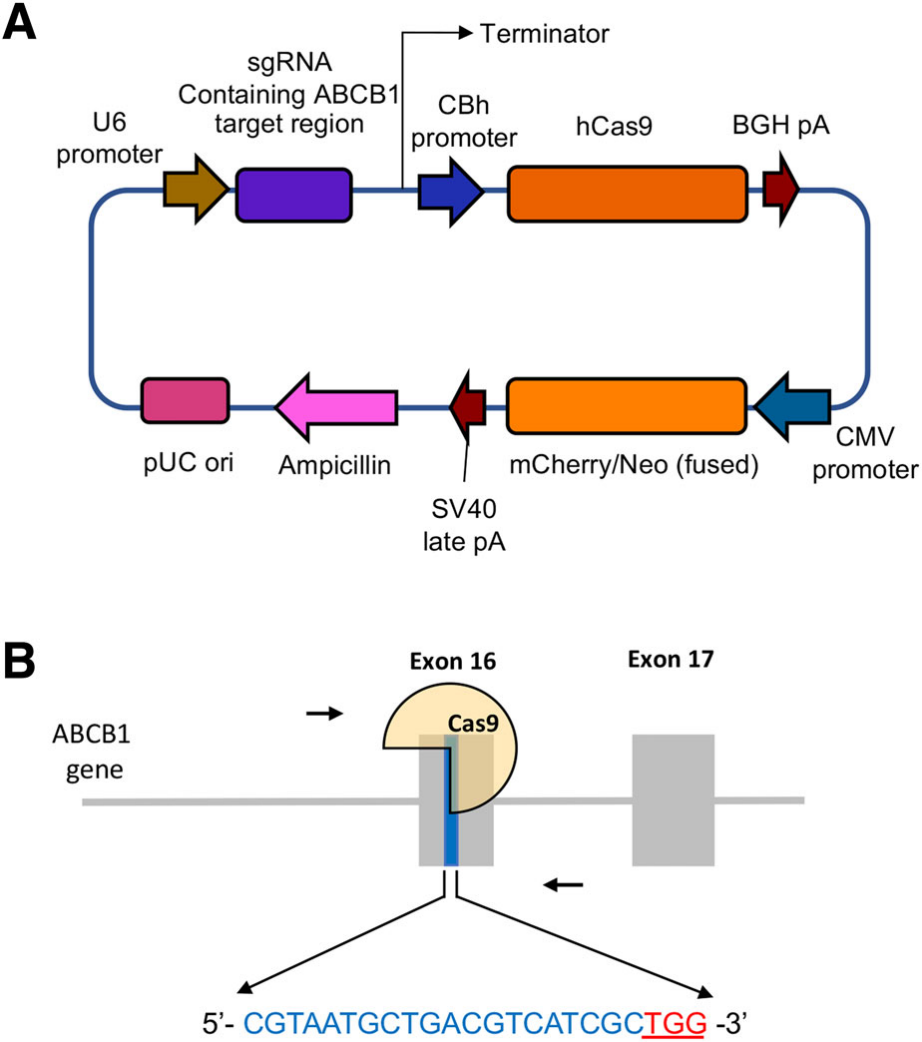
Schematic of the CRISPR‐Cas9 system used for *ABCB1* gene knockout. (A) The plasmid map of CRISPR/Cas9 vector. (B) The locations and sequences of the gRNA targeting the 16th exon of the *ABCB1* gene. The 20 bp target sequence for gRNA is shown in blue font, and the sequence of the protospacer adjacent motif (PAM) “TGG” designated for recognition by the Cas9 nuclease is highlighted in red with an underline

**FIGURE 2:**
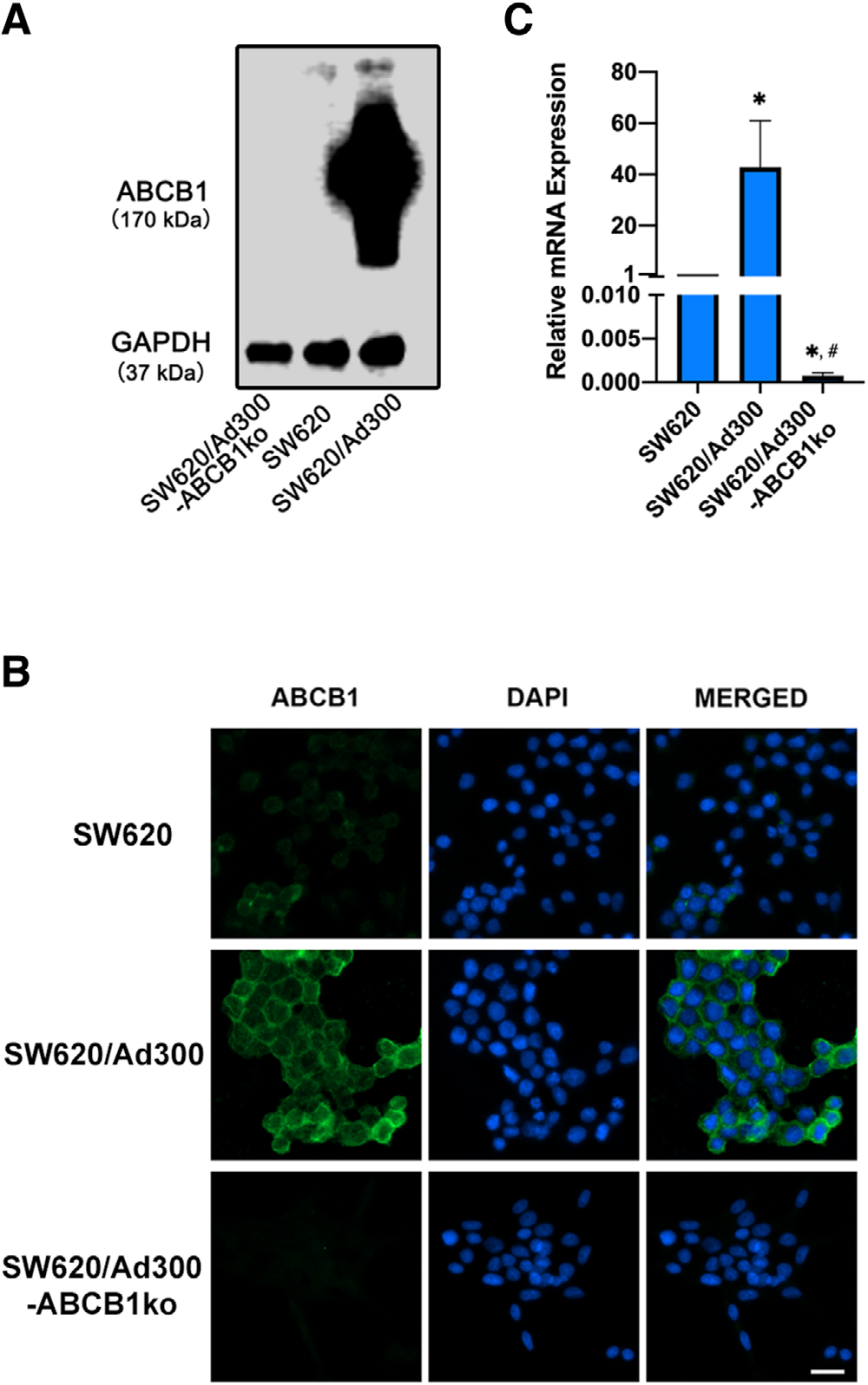
Confirmation of *ABCB1* knockout in SW620/Ad300 cells. (A) Western blotting results of ABCB1 protein expression level in cells. GAPDH was used as a loading control. (B) Immunofluorescence microscopic images showing ABCB1 expression by green fluorescence in cells. DAPI‐stained cell nuclei showed a blue color. The merged images were generated by combining the ABCB1 and DAPI fluorescence micrographs. Scale bar represented 20 μm. (C) mRNA expression levels of *ABCB1* gene measured by RT‐qPCR were normalized by the expression of *GAPDH* gene. Relative mRNA expression was presented as fold change versus SW620. * *p *< 0.05 compared to SW620. # *p *< 0.05 compared to SW620/Ad300. Columns and error bars represented mean values and standard deviation (SD) obtained from three independent measurements

The *ABCB1* gene knockout sublines of SW620/Ad300 cell line were established using a CRISPR/Cas9 system targeting exon 16 of *ABCB1* gene (Figure 1). The knockout of *ABCB1* gene in SW620/Ad300 cells was verified by the ABCB1 protein expression detected using Western blotting. As shown in Figure 2A, the acquired overexpression of ABCB1 protein was confirmed in SW620/Ad300 cells compared to the parental SW620 cells. The ABCB1 protein level was undetectable in SW620/Ad300‐ABCB1ko cells. Identical observations were shown in immunofluorescence results. The expression of ABCB1 on the cell membrane was much lower in the parental SW620 cells than in the drug‐resistant SW620/Ad300 cells, whereas the cells with *ABCB1* gene knockout were at an undetectable level by immunofluorescence staining (Figure 2B).

To further verify the change in gene expression by targeting *ABCB1* using the CRISPR/Cas9 system, the cDNA was synthesized from the mRNA of cells and amplified using specific primers by PCR reaction, which was quantified using SYBR labeling. The mRNA expression level of *ABCB1* was normalized to the reference gene *GAPDH*. As exhibited in Figure 2C, the *ABCB1* expression of SW620/Ad300 cells was upregulated approximately 40‐fold compared to the parental SW620 cells. *ABCB1* gene knockout by CRISPR/Cas9 system resulted in significant repression of *ABCB1* expression in SW620/Ad300 cells to a level of less than 1 of 1000 of the *ABCB1* expression in the parental SW620 cells. These data suggest that the SW620/Ad300‐ABCB1ko subline with stable *ABCB1* knockout were successfully established.

### 2.2 Drug resistance phenotype of ABCB1‐knockout MDR cells

**FIGURE 3:**
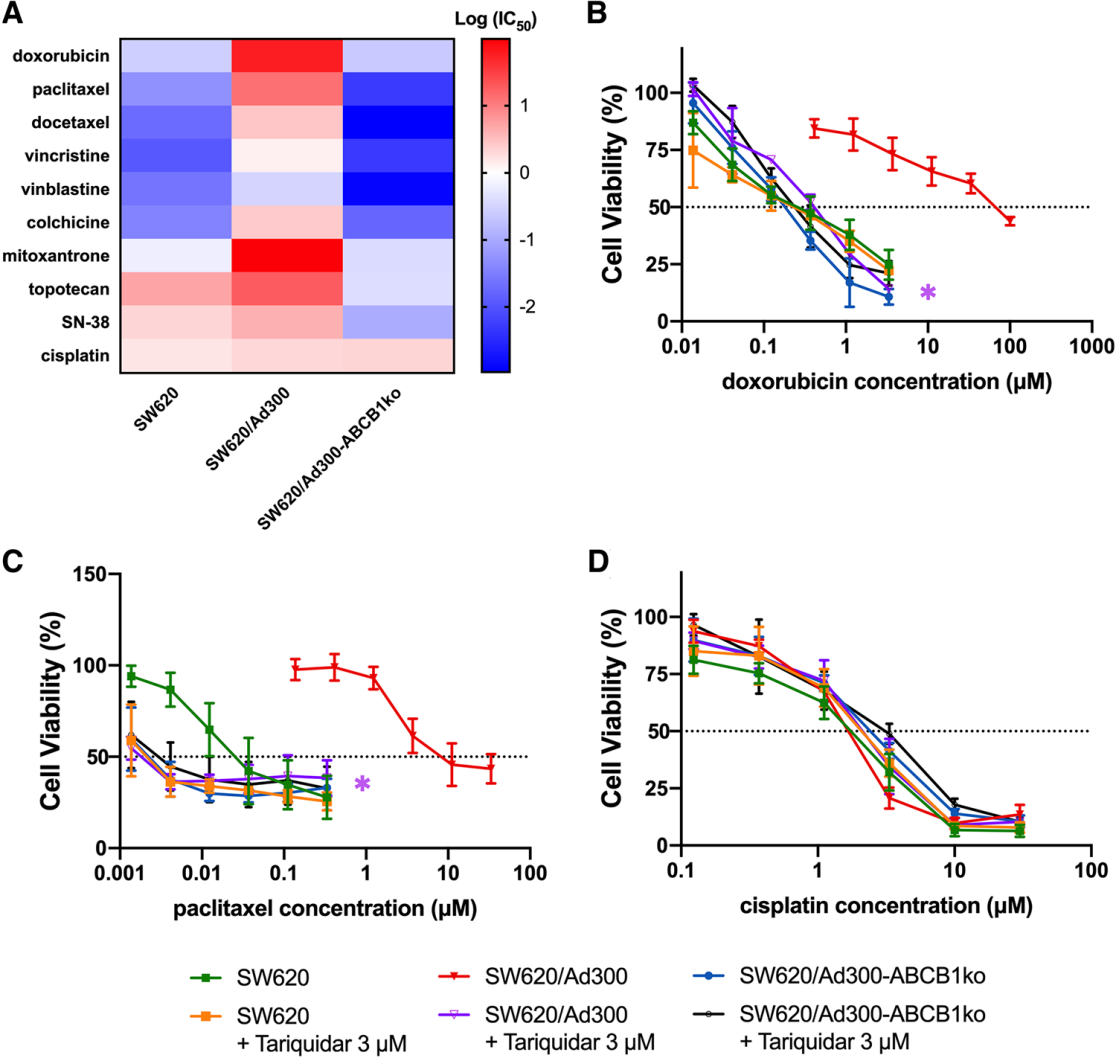
Drug resistance phenotype of MDR colorectal cancer cells with *ABCB1* knockout. (A) Heatmap of log IC_50_ values of anticancer drugs obtained from MTT assay against the utilized human colorectal cancer cell lines. The red color indicated higher logIC_50_ level, the white color indicated logIC_50 _= 0, and the blue color showed lower logIC_50_ level. (B‐D) Change of cell viability determined by MTT assay in response to various concentrations of doxorubicin (B), paclitaxel (C), and cisplatin (D), with or without the presence of 3 μM tariquidar. Data points with error bars displayed the average viability (%) ± SD obtained from at least three independent experiments performed in triplicate. The IC_50_ values were subjected to statistical analysis. The * labels were shown in the corresponding color to the figure legends. * refers to *p *< 0.05 comparing the group with 3 μM tariquidar to the group of the corresponding cell line without tariquidar

The drug resistance phenotypes of SW620, SW620/Ad300, and SW620/Ad300‐ABCB1ko cells were compared in Table 1 and Figure 3A. The SW620/Ad300 cells with ABCB1 overexpression induced by chronic doxorubicin selection exhibited 214.02‐fold resistance against doxorubicin compared to the parental SW620 cells. Significant cross‐resistance against doxorubicin, paclitaxel, docetaxel, vincristine, vinblastine, colchicine, mitoxantrone, and topotecan was observed in SW620/Ad300 cells. The MDR phenotype in SW620/Ad300 cells was significantly reversed by knockout of the *ABCB1* gene. By contrast, for cisplatin, which is not an ABCB1 substrate, the IC_50_ values were at a comparable level among SW620, SW620/A300, and SW620/Ad300‐ABCB1ko cells. To compare the effect of ABCB1 inhibitor on chemotherapeutic sensitivity in cells with and without *ABCB1* knockout, tariquidar, which is a third‐generation ABCB1 inhibitor that can inhibit the efflux function of ABCB1 and restore the intracellular drug accumulation,^20^ was selected for the reversal test. Doxorubicin and paclitaxel as representative substrate drugs of ABCB1, and cisplatin as a nonsubstrate drug, were picked for further reversal study. The concentration of tariquidar was selected at a nontoxic concentration that reduces cell viability by less than 15% through 72 h. While the response to cisplatin was consistent among the cell lines regardless of knockout of *ABCB1* or the presence of tariquidar (Figure 3D), tariquidar at 3 μM was capable to completely reverse the resistance to doxorubicin and paclitaxel in ABCB1 overexpressing SW620/Ad300 cells, but not in SW620 cells with trace level of ABCB1 expression (Figure 3B and C). The concentration‐response curves for doxorubicin and paclitaxel from *ABCB1* knockout cells remained unchanged with the addition of tariquidar and resembled closely to the ones from wildtype cells with the presence of 3 μM tariquidar. Identical findings were obtained from the reversal tests using another two ABCB1 inhibitors, tepotinib and verapamil, respectively. Both tepotinib and verapamil at 0.3 μM could reverse paclitaxel resistance in SW620/Ad300 cells but had no effects on *ABCB1* knockout cells, and the IC_50_ values for cisplatin remained consistent among the cell lines regardless of the presence of ABCB1 inhibitors (Supporting information Figure S2B and C). These results suggested that the abrogation of MDR in *ABCB1* gene knockout cells was majorly due to the loss of functional ABCB1 protein.

**TABLE 1:** Drug resistance spectrum of ABCB1 knockout cells

	IC_50_ ± SD (μM) (Resistant fold)		
Anticancer drug	SW620	SW620/Ad300	SW620/Ad300‐ABCB1ko
doxorubicin	0.275 ± 0.035 (1.00)	58.856 ± 4.977 (214.02)*	0.230 ± 0.031 (0.84)^#^
Paclitaxel	0.051 ± 0.013 (1.00)	13.460 ± 1.611 (263.92)*	0.005 ± 0.001 (0.10)^#^
Docetaxel	0.014 ± 0.002 (1.00)	2.763 ± 0.248 (197.36)*	0.001 ± 0.0001 (0.07)^#^
vincristine	0.011 ± 0.003 (1.00)	1.294 ± 0.038 (117.64)*	0.008 ± 0.002 (0.73)^#^
vinblastine	0.006 ± 0.003 (1.00)	0.311 ± 0.035 (51.83)*	0.001 ± 0.0004 (0.17)^#^
Colchicine	0.035 ± 0.007 (1.00)	2.608 ± 0.506 (74.51)*	0.016 ± 0.004 (0.46)^#^
mitoxantrone	0.632 ± 0.079 (1.00)	98.895 ± 1.53 (156.48)*	0.372 ± 0.018 (0.58)^#^
Topotecan	5.161 ± 0.429 (1.00)	19.088 ± 0.411 (3.70)*	0.391 ± 0.072 (0.08)*,^#^
SN‐38	2.105 ± 0.173 (1.00)	3.350 ± 0.487 (1.59)	0.106 ± 0.037 (0.05)*,^#^
Cisplatin	1.733 ± 0.222 (1.00)	2.034 ± 0.161 (1.17)	2.134 ± 0.121 (1.23)

IC_50_ refers to the concentration that reduces cell viability by 50% (mean ± SD). Resistance fold for SW620, SW620/Ad300, and SW620/Ad300‐ABCB1ko cells represents the IC_50_ value of an anticancer drug for SW620/Ad300 cells or SW620/Ad300‐ABCB1ko cells divided by that for SW620 cells.

Values in table are determined from at least three independent experiments conducted in triplicate.

* indicates *p *< 0.05 compared to IC_50_ value for SW620.

^#^ indicates *p *< 0.05 compared to IC_50_ value SW620/Ad300.

Slightly reduced IC_50_ values for the tested anticancer drugs except cisplatin were observed in SW620/Ad300‐ABCB1ko cells when compared to SW620 cells, however, most of these differences did not have statistical significance (Table 1). The variation in IC_50_ values between SW620 cells and SW620/Ad300‐ABCB1ko cells may be contributed by the low endogenous expression of ABCB1 in SW620 cells. This was further confirmed by *ABCB1* gene knockout in SW620 cells (verification of *ABCB1* knockout shown in Supporting information Figure S1A and B) and the follow‐up drug resistance spectrum analysis. As illustrated in Supporting information Figure S2A, the IC_50_ values for doxorubicin, paclitaxel, colchicine, vincristine, vinblastine, and mitoxantrone from SW620‐ABCB1ko cells were slightly lower than those from SW620 cells but closed to those from SW620/Ad300‐ABCB1ko cells, which supported the hypothesis that the endogenous ABCB1 expression accounts for the difference between SW620 cells and the *ABCB1* knockout cells in response to these drugs. Among the observed drug responses, the resistance to topotecan and SN‐38 from SW620/Ad300 cells was relatively low, with 3.7‐ and 1.59‐fold resistance, respectively, compared to SW620 cells. However, the SW620/Ad300‐ABCB1ko cells showed significantly enhanced sensitivity to topotecan and SN‐38 when comparing with SW620 as well as SW620/Ad300 cell lines, inferring that knockout of *ABCB1* gene may lead to changes in other biological molecules involved in the mechanism of action of topotecan and SN‐38. To verify whether this phenomenon was associated with alternation on ABCG2 or ABCC1 caused by CRISPR/Cas9‐mediated *ABCB1* knockout, the expression of ABCG2 and ABCC1 was measured in SW620, SW620/Ad300, and the ABCB1 knockout sublines. As shown in supporting information Figure S1C, the ABCG2 and ABCC1 expression remained at an undetectable level in SW620 and SW620/Ad300 cells after *ABCB1* gene knockout, indicating an alternative mechanism for the change of sensitivity to topotecan and SN‐38 upon loss of ABCB1.

### 2.3 Effect of ABCB1 knockout on [^3^H]‐paclitaxel accumulation and efflux in MDR colorectal cancer cells

**FIGURE 4:**
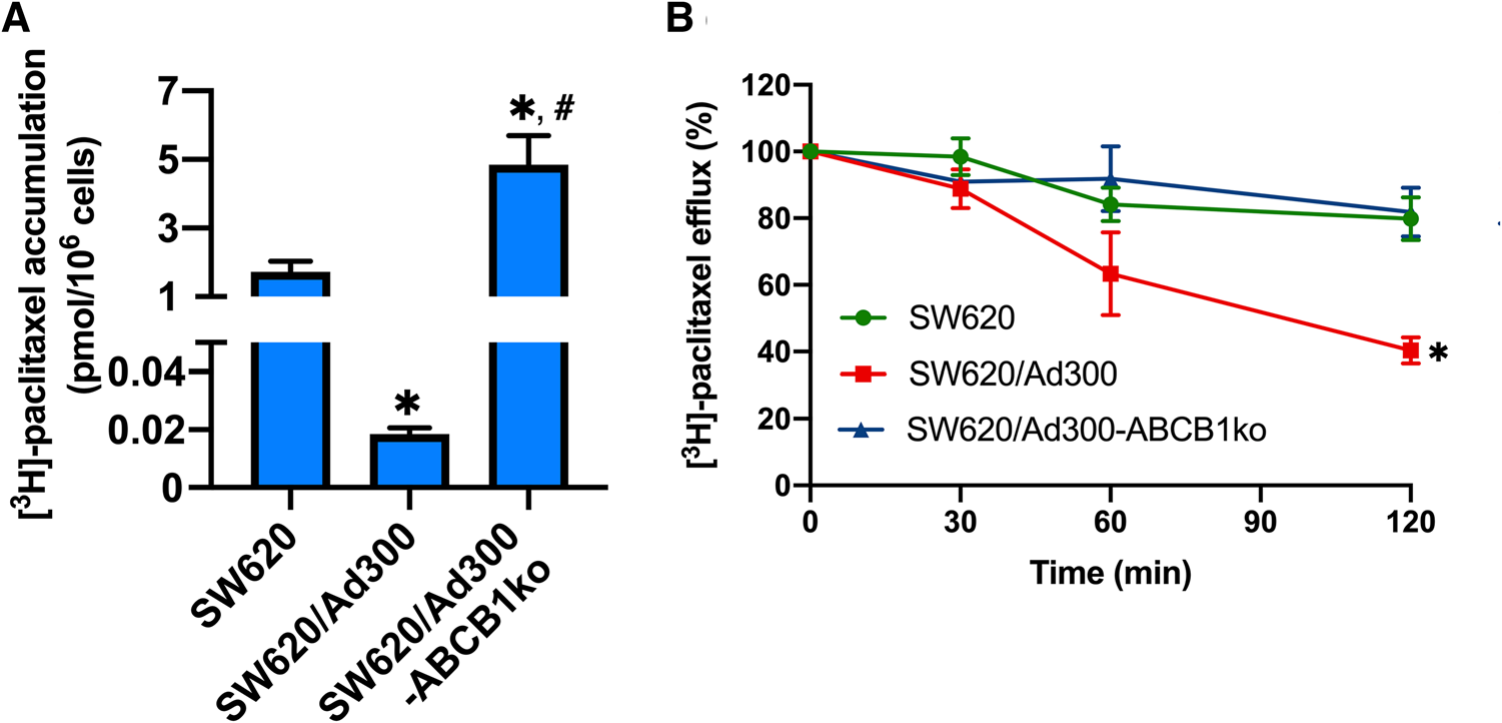
Effect of *ABCB1* gene knockout on intracellular accumulation and efflux of [^3^H]‐paclitaxel. (A) The intracellular accumulation of [^3^H]‐paclitaxel in SW620, SW620/Ad300, and SW620/Ad300‐ABCB1ko cells. * indicates *p *< 0.05 compared to SW620 cells; # indicates *p *< 0.05 compared to SW620/Ad300 cells. (B) The [^3^H]‐paclitaxel efflux activities of SW620, SW620/Ad300, and SW620/Ad300‐ABCB1ko cells. * indicates *p *< 0.05 compared to SW620 cells. Data represented the mean ± SD of three independent experiments performed in triplicate

The intracellular accumulation of ABCB1 substrate [^3^H]‐paclitaxel and the drug efflux activity were measured to examine the effect of *ABCB1* knockout on MDR colorectal cancer cells. As depicted in Figure 4A, the reduced intracellular [^3^H]‐paclitaxel level in SW620/A300 cells was restored by knockout of *ABCB1* to a level higher than that in SW620 cells. Besides, the [^3^H]‐paclitaxel efflux activity was mitigated in SW620/Ad300‐ABCB1ko cells compared to SW620/Ad300 cells, but there was no significant difference compared to SW620 cells (Figure 4B). These data suggested that *ABCB1* gene knockout using the CRISPR/Cas9 system was effective in restoring the intracellular level and decrease the efflux of ABCB1 substrate [^3^H]‐paclitaxel in ABCB1 overexpressing MDR colorectal cancer cells.

### 2.4 Effect of ABCB1 knockout on the growth and drug response of MDR colorectal cancer tumor spheroids

**FIGURE 5:**
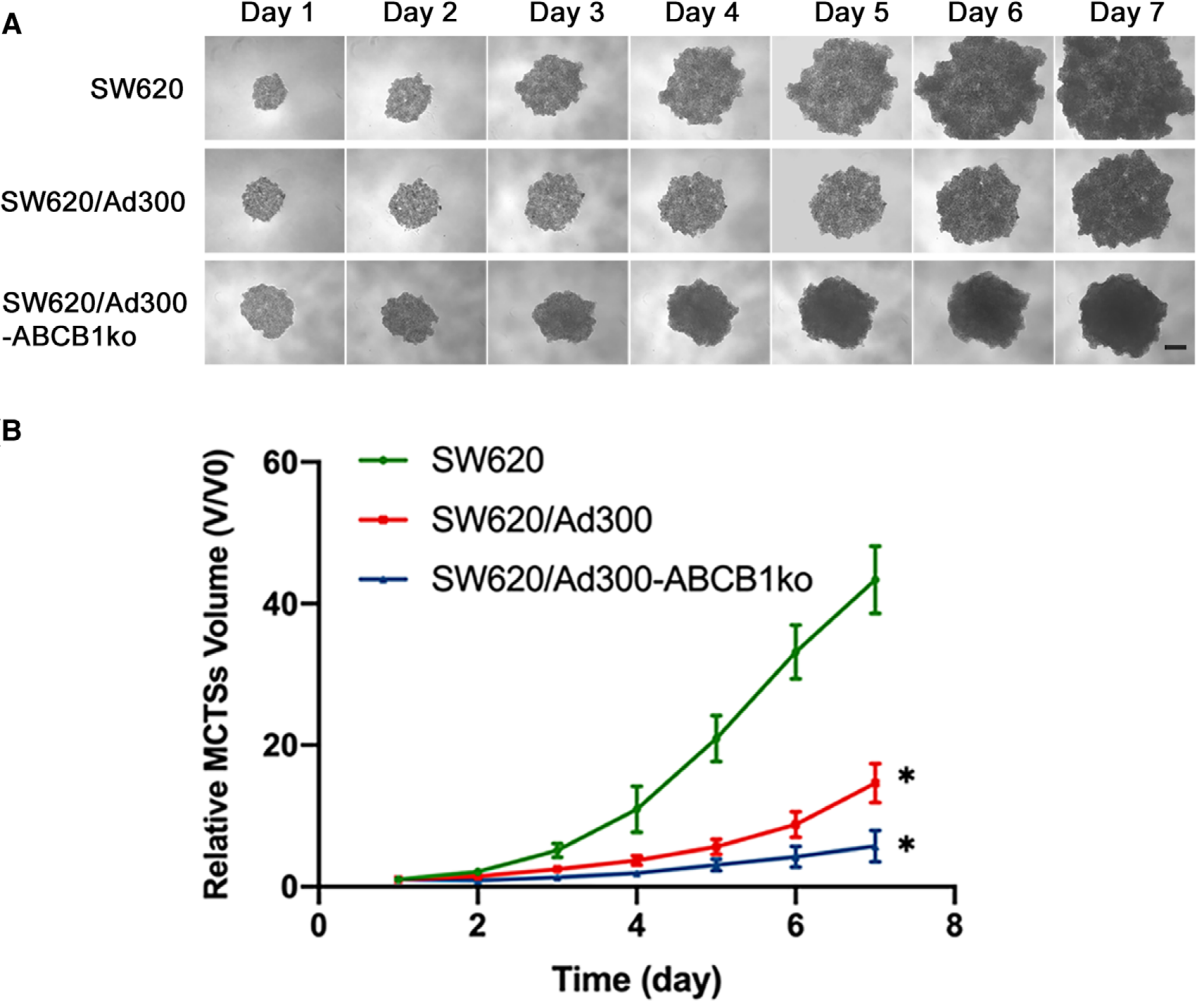
Effect of *ABCB1* gene knockout on morphology and growth of 3D multicellular tumor spheroids (MCTSs). (A) Representative images of the MCTSs grown from day 1 to 7 after seeding. Scale bar represented 200 μm. (B) Growth curve of MCTSs over 7 days. The volumes of MCTSs were normalized using the spheroid volume at day 1. Data points and error bars represented the mean and SD values obtained from three independent experiments performed with six replicates. * indicates *p *< 0.05 compared to SW620

To further investigate the effect of *ABCB1* knockout on the growth and drug sensitivity of MDR colorectal cancer cells in a tumor structure, the multicellular tumor spheroid (MCTS) model was used to mimic the natural biology of tumors. All cell lines tested were able to form tumor spheroids, but the spheroid morphology varied. The SW620/Ad300‐ABCB1ko spheroids maintained a morphology of loose aggregates as SW620 and SW620/Ad300 cells on day 1 postseeding, however, they showed increased compactness but a slower expansion in diameter from day 2 of culture (Figure 5A). While the growth rate of SW620/Ad300 spheroids was significantly lower than the parental SW620 spheroids, knockout of *ABCB1* led to a further decrease in MCTSs growth rate, but the decrease was not significant when comparing with SW620/Ad300 spheroids (Figure 5B). These results indicated that knockout of *ABCB1* may induce growth retardation and less aggressive expansion in MDR colorectal cancer MCTSs.

**FIGURE 6:**
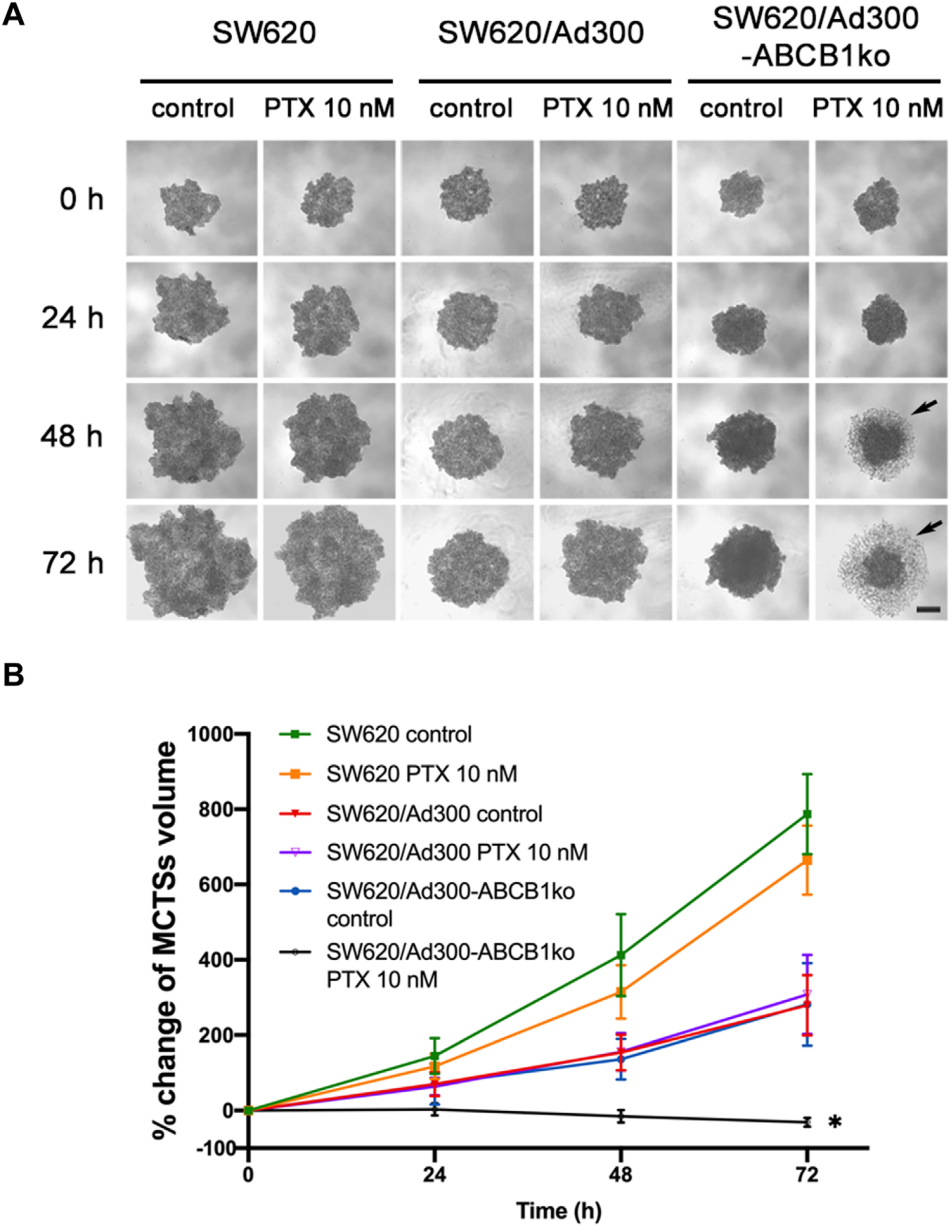
Effect of *ABCB1* gene knockout in cells on sensitivity to paclitaxel (PTX). (A) Representative images of the MCTSs treated with either vehicle control (culture media) or 10 nM paclitaxel at time points 0, 24, 48, and 72 h scale bar represented 200 μm. The loosely associated dead cells at the outer layer of spheroids were pointed by the black arrows. (B) Change of MCTSs volumes after treatment. Percentage of MCTSs volume was calculated by (spheroid volume–spheroid volume at timepoint‐0)/spheroid volume at timepoint‐0 × 100%. Data points and error bars showed the mean and SD obtained from three independent experiments performed with 4 replicates. * indicates *p *< 0.05 compared to the control group of the corresponding cell line

The drug sensitivity of *ABCB1* knockout spheroids was also tested. The ABCB1 substrate drug paclitaxel was selected for the toxicity assay against MCTSs. It has been reported that MCTSs could have higher resistance to chemotherapeutic agents than 2D monolayer cultured cells.^21^ Therefore, the concentration of paclitaxel at 10 nM, which was approximately two‐fold of the IC_50_ for paclitaxel obtained from SW620/Ad300‐ABCB1ko cells by MTT assay, was selected. As depicted in Figure 6, paclitaxel at 10 nM induced significant growth inhibition in SW620/Ad300‐ABCB1ko spheroids, whereas the growth of spheroids from SW620 and SW620/Ad300 cells was hardly affected by 10 nM paclitaxel. SW620/Ad300‐ABCB1ko spheroids treated with paclitaxel exhibited cell death and shedding from the outer zone (Figure 6A pointed by black arrows) and a decrease in the diameter of the cell aggregates (Figure 6B). A similar observation to that from SW620/Ad300‐ABCB1ko spheroids was that knockout of *ABCB1* also enhanced the toxicity of paclitaxel in SW620 spheroids (Supporting information Figure S1D and E). This further confirmed that the endogenous ABCB1 expression in SW620 cells accounted for the resistance to paclitaxel as well. The augmented responsiveness to paclitaxel in *ABCB1* knockout spheroids was consistent with the drug resistance phenotype observed from the MTT assay.

## 3 DISCUSSION

The ABCB1 expression is closely correlated with MDR in colorectal cancer, therefore, various approaches to suppress ABCB1 have been developed extensively investigated. As the outcomes for applications of ABCB1 inhibitors in clinical settings have been disappointing, novel strategies to surmount ABCB1‐mediated cancer MDR, such as gene therapy approaches, are gaining more attention.^18^ The novel CRISPR/Cas9 gene‐editing tool, which has greater precision than existing gene modifying technologies, has become more frequently used in cancer biological, oncological, and pharmaceutical research.^22^ The first published study, where this new technology was utilized to knockout the *ABCB1* gene, was carried out in Madin‐Darby canine kidney II (MDCK II) cells.^23^ In this study, the canine Abcb1 gene was completely knockout in MDCK II cells by transfected with the CRISPR/Cas9 plasmid vector, leading to the revocation of *ABCB1* transporting activity. Later, several studies with successful knockout of the *ABCB1* gene in human carcinoma cell lines have been reported. Liu et al. demonstrated that after silencing the *ABCB1* gene by CRISPR/Cas9 in osteosarcoma MDR cell lines, the drug resistance against ABCB1 substrate doxorubicin was reversed while the response to non‐ABCB1 substrate cisplatin was unchanged.^24^ Similar results were found by Yang et al. from two MDR cell lines KBV200 and HCT‐8/V, which gained ABCB1 overexpression by chronic exposure to vincristine. KBV200 and HCT‐8/V cells with knockout of *ABCB1* by CRISPR/Cas9 became significantly more sensitive to vincristine and doxorubicin whereas not to cisplatin, and had significant enhancement in accumulation of rhodamine123 as well as doxorubicin.^25^ Another acquired ABCB1 overexpressing cell line derived from human epithelial ovarian cancer cells by prolonged doxorubicin selection, termed A2780/ADR, was also found to restore sensitivity to doxorubicin after CRISPR/Cas9‐mediated *ABCB1* knockout was applied.^26^ These studies have demonstrated a general effect of restored sensitivity of ABCB1 substrate drug in MDR cancer cells after *ABCB1* knockout using CRISPR/Cas9 system, however, nothing was known regarding the variation in drug sensitivity of acquired MDR cell line with *ABCB1* knockout comparing to that of the parental cell lines from which the MDR cell lines originated. Besides, a broader spectrum of drug sensitivity after knockout of the *ABCB1* gene is yet to be evaluated in MDR colorectal cancer cells. Accordingly, the ABCB1 overexpressing MDR human colorectal cancer cell line SW620/Ad300 along with its parental SW620 cell line, were selected in the present study. A CRISPR/Cas9 system was used to knockout the *ABCB1* gene in MDR cells. Subsequently, the efficiency of the designed gene‐editing CRISPR/Cas9 system to knockout *ABCB1* gene was verified by the suppressed ABCB1 protein expression and mRNA expression levels of *ABCB1* gene in the knockout sublines.

After *ABCB1* gene knockout using CRISPR/Cas9 system in MDR SW620/Ad300 cells, cells showed enhanced sensitivity to ABCB1 substrate drugs, such as doxorubicin, paclitaxel, docetaxel, vincristine, vinblastine, colchicine, and mitoxantrone, but not to non‐ABCB1 substrate cisplatin. The increased intracellular accumulation of [^3^H]‐paclitaxel accumulation resulted from the decreased activity of paclitaxel efflux was observed, which was consistent with the findings in other human MDR cancer cell lines.^24^, ^25^, ^26^ The results that ABCB1 inhibitor tariquidar reversed doxorubicin and paclitaxel in MDR cells but not *ABCB1* knockout cells further verified that the abolishment of MDR in *ABCB1* knockout cells was predominantly contributed by the loss of functional ABCB1 protein. This was further confirmed by applying a first‐generation ABCB1 inhibitor verapamil and a newly discovered ABCB1 inhibitor tepotinib in supplementary reversal tests. From another dimension, the results from the reversal studies suggested that the *ABCB1* gene knockout cell lines can be useful in investigating whether ABCB1‐independent mechanisms are involved in the MDR reversal activities of ABCB1 inhibitors.

Comparing to the parental SW620 cell line, the IC_50_ values for ABCB1 substrate drugs obtained from SW620/Ad300‐ABCB1ko cells were even lower, albeit the differences were mostly insignificant. To confirm whether this is led by the low endogenous ABCB1 expression in SW620 cells, knockout of the *ABCB1* gene using the CRISPR/Cas9 system was further applied to SW620 cells. From the drug resistance spectrum of the cells, the effectiveness of common ABCB1 substrates, including doxorubicin, paclitaxel, docetaxel, vincristine, vinblastine, colchicine, and mitoxantrone, were negatively associated with the ABCB1 expression level in colorectal cancer cells. This was reflected by the considerable resistance from SW620/Ad300 cells, slight resistance from SW620 cells, and enhanced sensitivity from *ABCB1* knockout cells. As topotecan and SN‐38 are typical BCRP/ABCG2 substrates but less potent substrates of ABCB1, ABCB1 may not have a significant contribution to extrude topotecan and SN‐38 from MDR cells,^27^, ^28^ which was reflected by the low resistant folds for topotecan and SN‐38 from ABCB1 overexpressing SW620/Ad300 cells. Interestingly, although resistance to topotecan and SN‐38 were low in SW620/Ad300 cells, the SW620/Ad300‐ABCB1ko cells showed significantly enhanced sensitivity to topotecan and SN‐38 when comparing with SW620 as well as SW620/Ad300 cell lines, and similar results were observed from SW620‐ABCB1ko cells. This phenomenon indicated that possible changes in molecules involved in the mechanism of action of topotecan and SN‐38 might be driven by the CRISPR/Cas9 vector targeting the *ABCB1* gene, which requires further study to elucidate.

Considering the limitation of 2D monolayer cell culture that the monolayer culture cells do not reflect the natural structures of tumors and the cell‐cell or cell‐extracellular environment interactions, and that the drug diffusion patterns are altered,^29^ the 3D MCTS model was used to mimic the tumor growth and sensitivity to anticancer drug in vivo. The morphology of loose aggregates from SW620 spheroids was consistent with previous reports.^30^, ^31^ A significantly slower growth rate was observed from SW620/Ad300 MCTSs compared to the parental SW620 spheroids, which was similar to previous studies that demonstrated decreased growth rates in doxorubicin‐resistant cancer cells or tumor xenograft model.^32^, ^33^ It has been commonly reported that the MDR phenotype of cancer cells is associated with slower cell proliferation, however, the exact mechanism correlating MDR and slow tumor growth remains uncertain. Slower growth of ABCB1 overexpressing cancer cells has been reported while this phenomenon could be contributed by multiple factors such as hypoxic conditions of cells or oxygen gradients in spheroids, alteration in expression of fibroblast growth factor and vascular endothelial growth factor, and intercellular transfer of ABCB1 within a heterogeneous tumor.^33^, ^34^ Some of the factors, like cell hypoxia, may induce retardative cell proliferation via an ABCB1‐independent mechanism.^35^ The slower growth of SW620/Ad300 spheroids compared to the parental SW620 spheroids may be partially accounted by ABCB1 overexpression and additional mechanisms causing growth arrest may exist, which could explain the observation that SW620/Ad300‐ABCB1ko spheroids retained a low growth rate like SW620/Ad300 spheroids.

Although an inversed correlation between ABCB1 expression and cancer cell growth rate has been commonly known, deficiency in ABCB1 protein expression was shown to result in further restrained tumor growth in colitis‐associated colorectal cancer mouse model, which may be related to change in pathways involved in inflammatory response.^36^ In this study, the growth curves obtained from MCTSs also revealed a slightly slower growth rate and less invasive expansion in MDR colon tumor spheroids led by ABCB1 silencing. However, the difference in growth rate was not significant when comparing SW620/Ad300‐ABCB1ko with SW620/Ad300 spheroids, possibly due to less significant involvement of inflammatory mediators in biological activities of SW620/Ad300 cells as the SW620 cell line is not derived from colitis‐associated colorectal cancer tissue. The morphology of tumor spheroid also reflected the restored drug sensitivity mediated by *ABCB1*‐targeting CRISPR/Cas9 gene editing. The elevated sensitivity to paclitaxel from MCTSs generated from SW620/Ad300‐ABCB1ko and SW620‐ABCB1ko cells were shown by loss of integrity and loosely associated dead cells at the outer layer after 48 and 72 h of exposure, which was consistent with the appearance of paclitaxel‐treated MCTSs from SW620/Ad300 cells reported by Gao et al.^37^

Overall, the present study suggests that the CRISPR/Cas9 system targeting the *ABCB1* gene can be an effective approach to overcome ABCB1‐mediated MDR in colorectal cancer cells. Compared to small molecule inhibitors, the novel CRISPR/Cas9‐gene editing approach has a great potential for future personalized therapy in treating ABCB1‐associated MDR colorectal cancer with an advantage to avoid drug‐drug interaction and toxicity from combinational chemotherapy. However, currently, the utilization of the CRISPR/Cas9 gene‐modifying technique in cancer treatment is limited in cell‐based or tumor xenograft animal‐based research.^38^ Efficient delivery of the CRISPR/Cas9 system in vivo and the associated ethical issues remain the major challenges to be solved before promoting to clinical application. The established *ABCB1* knockout colorectal cancer cell lines can be useful as models for studying ABCB1‐independent MDR mechanisms and discovering novel therapeutic approaches in future research to provide clues for overcoming multidrug resistance in colorectal cancer.

## 4 MATERIALS AND METHODS

### 4.1 Chemicals and reagents

Doxorubicin, paclitaxel, vincristine, vinblastine, colchicine, topotecan, tariquidar, verapamil, formaldehyde, Triton X‐100, 3‐(4, 5‐dimethylthiazol‐yl)‐2, 5‐diphenyltetrazolium bromide (MTT), agarose, and the mouse monoclonal antibody (mAb) for ABCB1 (clone F4) were purchased from Sigma Chemical Co. (St. Louis, MO). Geneticin (G418), mitoxantrone, SN‐38, and cisplatin were obtained from Enzo Life Sciences (Farmingdale, NY). Tepotinib was provided by Chemie Tek (Indianapolis, IN). The radiolabeled drug [^3^H]‐paclitaxel (31 Ci/mmol) was ordered from Moravek Biochemicals, Inc. (Brea, CA). Fetal bovine serum (FBS), Dulbecco's modified Eagle's medium (DMEM), and 0.25% trypsin‐EDTA were ordered from Corning Inc. (New York, NY). The horseradish peroxidase‐conjugated secondary antibody (anti‐mouse) was obtained from Cell Signaling Technology (Danvers, MA). The mouse mAb for glyceraldehyde phosphate dehydrogenase (GAPDH), phosphate buffer saline (PBS), dimethyl sulphoxide (DMSO), the Alexa Fluor 488‐labeled secondary antibody (anti‐mouse), 4,6‐diamidino‐2‐phenylindole (DAPI), and other reagents were ordered from Thermo Fisher Scientific Inc. (Rockford, IL).

### 4.2 Cell lines and cell culture

The human colon adenocarcinoma SW620 and SW620/Ad300 cell lines were kind gifts from Dr. Susan E. Bates (Columbia University, New York, NY) and Dr. Robert W. Robey (NCI, NIH, Bethesda, MD). The drug‐induced ABCB1 overexpressing SW620/Ad300 cell line was derived from the parental SW620 cell line after prolonged exposure to gradually increase the concentration of doxorubicin up to 300 ng/mL.^39^ All cell lines were cultured in a 37℃ humidified incubator containing 5% CO_2_. The culture medium was 10% FBS‐supplemented DMEM supplemented with the addition of 100 units/mL penicillin/streptomycin.

### 4.3 Knockout of ABCB1 gene in SW620/Ad300 cells

A CRISPR/Cas9 system was used to construct the *ABCB1* gene knockout subline of SW620/Ad300 cell line. The custom‐designed mammalian CRISPR vector was purchased from VectorBuilder Inc. (Chicago, IL). The gRNA of the vector targeting human *ABCB1* gene contains a specific 20 bp guide sequence of 5′‐ CGTAATGCTGACGTCATCGC‐3′ selected from exon 16 of the human *ABCB1*. Transfection of the *ABCB1* targeting vector into SW620/Ad300 cells was performed using Fugene6 transfection reagent (Promega, Madison, WI) according to the manufacturer's instructions. Briefly, SW620/Ad300 cells were seeded in 100 mm dishes with 1 × 10^6^ cells per dish and cultured overnight in DMEM with 10% FBS without antibiotics. Then, 8 μg of plasmid DNA was prepared in 376 μL of Opti‐MEM medium and mixed with 24 μL of Fugene6 reagent. After a 30‐min incubation at room temperature, the complex was mixed into the cell culture medium and incubated with the cells in a culture incubator for 48 h. At the end of incubation, the transfected cells were rinsed with PBS then incubated with the selection medium containing 1.5 mg/mL G418 for 14 days with the medium changed every 3 days. Nontransfected cells were used as negative controls for the selection process. Single colonies of survived cells were obtained using the limited dilution method and expanded for further study. The knockout of *ABCB1* was further verified by measuring protein expression and mRNA expression levels using Western blotting and reverse transcription‐quantitative PCR and immunofluorescence analysis, respectively.

### 4.4 Western blotting

Cells, after two times of rinse with ice‐cold PBS, were lysed by 20‐min incubation with a modified NP‐40 lysis buffer on ice. The cell lysates were collected from the supernatant generated after 20‐min centrifugation with a speed of 12,000 ×*g* at 4°C. The protein concentration was quantified using Pierce BCA Protein Assay Kit (Thermo Fisher Scientific Inc.). An equal amount of total cell protein was loaded for SDS‐polyacrylamide gel electrophoresis (SDS‐PAGE) and electrotransferred to a polyvinylidene difluoride (PVDF) membrane. A 2 h blocking process for the PVDF membrane using 5% nonfat dried milk was followed before incubation in primary antibodies (1:1000 dilution) of ABCB1 and GAPDH overnight at 4°C. At the end of incubation with the primary antibodies, the PVDF membrane was washed thoroughly using Tris‐buffered saline with 0.1% Tween‐20 (TBST) and incubated in HRP‐linked secondary antibody (1:1000 dilution) for 2 h at room temperature. Visualization of the protein bands was conducted using enhanced chemiluminescence.

### 4.5 Reverse transcription‐quantitative PCR (RT‐qPCR)

Total mRNA of cells was extracted using Trizol reagent in accordance with the manufacturer's protocol. Total RNA concentrations and purity were determined by the optical density (OD) at 260 nm and the ratio of OD260nm/OD280nm. Reverse transcription was performed for cDNA synthesis. Quantitative gene analysis was performed using the fluorescent dye SYBR Select Master Mix (Applied Biosystems, Foster City, CA). The primer sequences for *ABCB1* gene were 5′‐ACGTCATCGCTGGTTTCGAT‐3′ (forward) and 5′‐ CTGCATTGTGACAAGTTTGAAGT‐3′ (reverse). The primer sequences for GAPDH gene were 5′‐CCTCAAGATCATCAGCAATGCC‐3′ (forward) and 5′‐TCTAGACGGCAGGTCAGGTC‐3′ (reverse). The PCR reactions were conducted in Aria Mx Real‐Time PCR System (Agilent Technologies, Santa Clara, CA). The mRNA expression of *ABCB1* gene was quantified using the delta‐delta *Ct* method and normalized by the expression of GAPDH gene.

### 4.6 Immunofluorescence analysis

Immunofluorescence analysis was carried out to further confirm the ABCB1 expression change in the knockout cells. Cells were prepared for antibody binding by undergoing fixation, permeabilization, and serum blocking processes as previously described.^37^ Cells were then incubated with the monoclonal antibody against ABCB1 (1:1000 dilution) overnight at 4°C followed by incubation with Alexa Flour 488‐linked secondary antibody (1:1000 dilution) for 2 h at room temperature. The nuclei of cells were visualized by counterstaining using DAPI. The EVOS FL Auto Imaging System (Thermo Fisher Scientific Inc.) was used for capturing images with immunofluorescence staining.

### 4.7 Cytotoxicity and reversal test

The MTT cytotoxicity assay was performed to investigate the drug resistance phenotype of the *ABCB1* knockout cells. A number of ABCB1 substrate drugs were tested, including doxorubicin, paclitaxel, docetaxel, vincristine, colchicine, mitoxantrone, topotecan, and SN‐38.^40^, ^41^ Cisplatin was used as a non‐ABCB1 substrate control.^42^ A total of 7000 cells were seeded per well into 96‐well plates and cultured overnight. Then, cells were treated with different concentrations of anticancer drugs for 72 h. At the end of treatment, the cell viability was determined by MTT colorimetric assay following the protocol described previously^43^ and the concentration‐response curve was graphed for calculation of the half‐maximal inhibitory concentration (IC_50_). In reversal tests, cells were treated with varying concentrations of anticancer drugs with or without the presence of 3 μM tariquidar, which was added 2 h before the treatment started. MTT assay was conducted at the end of the treatment to measure the reversal effect of tariquidar. A first‐generation ABCB1 inhibitor verapamil and a newly discovered ABCB1 inhibitor tepotinib,^44^ were used for supplementary reversal tests in this study.

### 4.8 [^3^H]‐paclitaxel accumulation and efflux assay

The accumulation levels of [^3^H]‐paclitaxel in cells and the drug efflux activity were compared among cells with or without *ABCB1* knockout. Cells were seeded 1 × 10^5^ cells per well into 24‐well plates and were allowed to settle by overnight culture. Then, cells were incubated in a medium containing 10 nM [^3^H]‐paclitaxel at 37°C for 2 h. When the incubation was completed, cells were washed with ice‐cold PBS then incubated in [^3^H]‐paclitaxel‐free medium. Cells were harvested at four time points (0, 30, 60, 120 min) and transferred into scintillation fluid. The measurement of radioactivity was carried out using the Tri‐Carb liquid scintillation counter (Packard Instrument Inc., Chicago, IL). The intracellular [^3^H]‐paclitaxel level at time point 0 was used to determine the accumulation of [^3^H]‐paclitaxel, and the change in intracellular [^3^H]‐paclitaxel levels among time points was used for investigating the drug efflux activity.

### 4.9 Growth and drug sensitivity of 3D MCTSs

Cells were seeded into 1% agarose‐coated 96‐well plates in a density of 500 cells per well. The images of the MCTSs were taken with an inverted phase‐contrast microscope and the diameters of the MCTSs were measured daily for up to 7 days’ culture with medium changed every third day. The sensitivity of MCTSs to paclitaxel was also investigated. The MCTSs were treated with 10 nM paclitaxel at 48 h postseeding of the cells when the MCTS aggregates formed to approximately 300 to 400 μm in diameter. The images of the MCTSs were taken and the diameters were measured at time points 0, 24, 48, and 72 h.

### 4.10 Statistical analysis

Statistical analysis was carried out in the GraphPad Prism 8 software (GraphPad Software, La Jolla, CA). Comparisons between mean values of multiple groups were carried out using one‐way ANOVA and the subsequent Tukey's post‐hoc test. Repeated measures two‐way ANOVA and the subsequent Tukey's post‐hoc test was performed to compare the differences among the groups with measurements at different time points in [^3^H]‐paclitaxel efflux analysis and MCTSs‐based assays. A criterion of *p* value less than 0.05 is set for statistical significance.

## CONFLICTS OF INTEREST

Author Zhe‐Sheng Chen is the Editor Board Member of MedComm. Author Zhe‐Sheng Chen was not involved in the journal's review of, or decisions related to, this manuscript. The other authors declare no conflict of interest.

## ETHICS STATEMENT

Not applicable.

## AUTHOR CONTRIBUTIONS

Z.N.L. and Z.S.C. conceived the project and designed the experiments. Z.N.L., Q.X.T., and Z.S.C. wrote and revised the manuscript. Z.N.L., Q.X.T., Z.X.W., F.F.P., and P.S. performed the experiments. Z.N.L., Q.X.T., and Z.X.W. analyzed and interpreted the experimental data. J.N.D.W. and Z.S.C. supervised the study.

## Supporting information

SUPPORTING INFORMATION

## Data Availability

The data that support the findings of this study are available from the corresponding author upon reasonable request.

## References

[1] Labianca R , Beretta GD , Kildani B , et al. Colon cancer. Crit Rev Oncol Hematol. 2010;74(2):106‐133. 20138539 10.1016/j.critrevonc.2010.01.010

[2] McQuade RM , Stojanovska V , Bornstein JC , Nurgali K . Colorectal cancer chemotherapy: the evolution of treatment and new approaches. Curr Med Chem. 2017;24(15):1537‐1557. 28079003 10.2174/0929867324666170111152436

[3] Vargas GM , Sheffield KM , Parmar AD , et al. Trends in treatment and survival in older patients presenting with stage IV colorectal cancer. J Gastrointest Surg. 2014;18(2):369‐377. 24234244 10.1007/s11605-013-2406-z PMC3960341

[4] Dehal A , Graff‐Baker AN , Vuong B , et al. Neoadjuvant chemotherapy improves survival in patients with clinical T4b colon cancer. J Gastrointest Surg. 2018;22(2):242‐249. 28933016 10.1007/s11605-017-3566-z

[5] Singh MS , Tammam SN , Boushehri MAS , Lamprecht A . MDR in cancer: Addressing the underlying cellular alterations with the use of nanocarriers. Pharmacol Res. 2017;126:2‐30. 28760489 10.1016/j.phrs.2017.07.023

[6] Hu T , Li Z , Gao CY , Cho CH . Mechanisms of drug resistance in colon cancer and its therapeutic strategies. World J Gastroenterol. 2016;22(30):6876‐6889. 27570424 10.3748/wjg.v22.i30.6876 PMC4974586

[7] Kartal‐Yandim M , Adan‐Gokbulut A , Baran Y . Molecular mechanisms of drug resistance and its reversal in cancer. Crit Rev Biotechnol. 2016;36(4):716‐726. 25757878 10.3109/07388551.2015.1015957

[8] Lee AJ , Endesfelder D , Rowan AJ , et al. Chromosomal instability confers intrinsic multidrug resistance. Cancer Res. 2011;71(5):1858‐1870. 21363922 10.1158/0008-5472.CAN-10-3604 PMC3059493

[9] Holohan C , Van Schaeybroeck S , Longley DB , Johnston PG . Cancer drug resistance: an evolving paradigm. Nat Rev Cancer. 2013;13(10):714‐726. 24060863 10.1038/nrc3599

[10] Hu T , Wang L , Zhang L , et al. Sensitivity of apoptosis‐resistant colon cancer cells to tanshinones is mediated by autophagic cell death and p53‐independent cytotoxicity. Phytomedicine. 2015;22(5):536‐544. 25981919 10.1016/j.phymed.2015.03.010

[11] Kozovska Z , Gabrisova V , Kucerova L . Colon cancer: cancer stem cells markers, drug resistance and treatment. Biomed Pharmacother. 2014;68(8):911‐916. 25458789 10.1016/j.biopha.2014.10.019

[12] Mohammad IS , He W , Yin L . Understanding of human ATP binding cassette superfamily and novel multidrug resistance modulators to overcome MDR. Biomed Pharmacother. 2018;100:335‐348. 29453043 10.1016/j.biopha.2018.02.038

[13] International Transporter Consortium , Giacomini KM , Huang SM , et al. Membrane transporters in drug development. Nat Rev Drug Discov. 2010;9(3):215‐236. 20190787 10.1038/nrd3028 PMC3326076

[14] Fu D , Arias IM . Intracellular trafficking of P‐glycoprotein. Int J Biochem Cell Biol. 2012;44(3):461‐464. 22212176 10.1016/j.biocel.2011.12.009 PMC3288648

[15] Meschini S , Calcabrini A , Monti E , et al. Intracellular P‐glycoprotein expression is associated with the intrinsic multidrug resistance phenotype in human colon adenocarcinoma cells. Int J Cancer. 2000;87(5):615‐628. 10925353

[16] Zhang H , Xu H , Ashby CR, Jr. , Assaraf YG , Chen ZS , Liu HM . Chemical molecular‐based approach to overcome multidrug resistance in cancer by targeting P‐glycoprotein (P‐gp). Med Res Rev. 2021;41(1):525‐555. 33047304 10.1002/med.21739

[17] Lo Y‐L , Liu Y . Reversing multidrug resistance in Caco‐2 by silencing MDR1, MRP1, MRP2, and BCL‐2/BCL‐xL using liposomal antisense oligonucleotides. PLoS One. 2014;9(3):e90180. 24637737 10.1371/journal.pone.0090180 PMC3956467

[18] Lage H . Gene therapeutic approaches to overcome ABCB1‐mediated drug resistance. Recent Results Cancer Res. 2016;209:87‐94. 28101689 10.1007/978-3-319-42934-2_6

[19] Montazami N , Kheir Andish M , Majidi J , et al. siRNA‐mediated silencing of MDR1 reverses the resistance to oxaliplatin in SW480/OxR colon cancer cells. Cell Mol Biol (Noisy‐le‐grand). 2015;61(2):98‐103. 26025411

[20] Fox E , Bates SE . Tariquidar (XR9576): a P‐glycoprotein drug efflux pump inhibitor. Expert Rev Anticancer Ther. 2007;7(4):447‐459. 17428165 10.1586/14737140.7.4.447

[21] Han SJ , Kwon S , Kim KS . Challenges of applying multicellular tumor spheroids in preclinical phase. Cancer Cell Int. 2021;21(1):152. 33663530 10.1186/s12935-021-01853-8 PMC7934264

[22] Tian X , Gu T , Patel S , Bode AM , Lee M‐H , Dong Z . CRISPR/Cas9—an evolving biological tool kit for cancer biology and oncology. NPJ Precision Oncology. 2019;3(1):1‐8. 30911676 10.1038/s41698-019-0080-7 PMC6423228

[23] Simoff I , Karlgren M , Backlund M , et al. Complete knockout of endogenous Mdr1 (Abcb1) in MDCK cells by CRISPR‐Cas9. J Pharm Sci. 2016;105(2):1017‐1021. 26869442 10.1016/S0022-3549(15)00171-9

[24] Liu T , Li Z , Zhang Q , et al. Targeting ABCB1 (MDR1) in multi‐drug resistant osteosarcoma cells using the CRISPR‐Cas9 system to reverse drug resistance. Oncotarget. 2016;7(50):83502. 27835872 10.18632/oncotarget.13148 PMC5347784

[25] Yang Y , Qiu JG , Li Y , et al. Targeting ABCB1‐mediated tumor multidrug resistance by CRISPR/Cas9‐based genome editing. Am J Transl Res. 2016;8(9):3986‐3994. 27725879 PMC5040697

[26] Norouzi‐Barough L , Sarookhani M , Salehi R , Sharifi M , Moghbelinejad S . CRISPR/Cas9, a new approach to successful knockdown of ABCB1/P‐glycoprotein and reversal of chemosensitivity in human epithelial ovarian cancer cell line. Iran J Basic Med Sci. 2018;21(2):181‐187. 29456815 10.22038/IJBMS.2017.25145.6230 PMC5811757

[27] Mo W , Zhang JT . Human ABCG2: structure, function, and its role in multidrug resistance. Int J Biochem Mol Biol. 2012;3(1):1‐27. 22509477 PMC3325772

[28] Gupta A , Dai Y , Vethanayagam RR , et al. Cyclosporin A, tacrolimus and sirolimus are potent inhibitors of the human breast cancer resistance protein (ABCG2) and reverse resistance to mitoxantrone and topotecan. Cancer Chemother Pharmacol. 2006;58(3):374‐383. 16404634 10.1007/s00280-005-0173-6

[29] Kapalczynska M , Kolenda T , Przybyla W , et al. 2D and 3D cell cultures—a comparison of different types of cancer cell cultures. Arch Med Sci. 2018;14(4):910‐919. 30002710 10.5114/aoms.2016.63743 PMC6040128

[30] Riedl A , Schlederer M , Pudelko K , et al. Comparison of cancer cells in 2D vs 3D culture reveals differences in AKT‐mTOR‐S6K signaling and drug responses. J Cell Sci. 2017;130(1):203‐218. 27663511 10.1242/jcs.188102

[31] Zoetemelk M , Rausch M , Colin DJ , Dormond O , Nowak‐Sliwinska P . Short‐term 3D culture systems of various complexity for treatment optimization of colorectal carcinoma. Sci Rep. 2019;9(1):1‐14. 31068603 10.1038/s41598-019-42836-0 PMC6506470

[32] Wosikowski K , Schuurhuis D , Kops GJ , Saceda M , Bates SE . Altered gene expression in drug‐resistant human breast cancer cells. Clin Cancer Res. 1997;3(12 Pt 1):2405‐2414. 9815641

[33] Levchenko A , Mehta BM , Niu X , et al. Intercellular transfer of P‐glycoprotein mediates acquired multidrug resistance in tumor cells. Proc Natl Acad Sci USA. 2005;102(6):1933‐1938. 15671173 10.1073/pnas.0401851102 PMC545583

[34] Wartenberg M , Donmez F , Ling FC , Acker H , Hescheler J , Sauer H . Tumor‐induced angiogenesis studied in confrontation cultures of multicellular tumor spheroids and embryoid bodies grown from pluripotent embryonic stem cells. FASEB J. 2001;15(6):995‐1005. 11292660 10.1096/fj.00-0350com

[35] Chen J , Ding Z , Peng Y , et al. HIF‐1alpha inhibition reverses multidrug resistance in colon cancer cells via downregulation of MDR1/P‐glycoprotein. PLoS One. 2014;9(6):e98882. 24901645 10.1371/journal.pone.0098882 PMC4047061

[36] Hennenberg EM , Eyking A , Reis H , Cario E . MDR1A deficiency restrains tumor growth in murine colitis‐associated carcinogenesis. PLoS One. 2017;12(7):e0180834. 28686677 10.1371/journal.pone.0180834 PMC5501609

[37] Gao HL , Gupta P , Cui Q , et al. Sapitinib reverses anticancer drug resistance in colon cancer cells overexpressing the ABCB1 transporter. Original Research. Front Oncol. 2020;10(2258):574861. 33163405 10.3389/fonc.2020.574861 PMC7581728

[38] Jiang C , Meng L , Yang B , Luo X . Application of CRISPR/Cas9 gene editing technique in the study of cancer treatment. Clin Genet. 2020;97(1):73‐88. 31231788 10.1111/cge.13589

[39] Lai GM , Chen YN , Mickley LA , Fojo AT , Bates SE . P‐glycoprotein expression and schedule dependence of adriamycin cytotoxicity in human colon carcinoma cell lines. Int J Cancer. 1991;49(5):696‐703. 1682280 10.1002/ijc.2910490512

[40] Hodges LM , Markova SM , Chinn LW , et al. Very important pharmacogene summary: ABCB1 (MDR1, P‐glycoprotein). Pharmacogenet Genomics. 2011;21(3):152‐161. 20216335 10.1097/FPC.0b013e3283385a1c PMC3098758

[41] Tagen M , Zhuang Y , Zhang F , et al. P‐glycoprotein, but not multidrug resistance protein 4, plays a role in the systemic clearance of irinotecan and SN‐38 in mice. Drug Metab Lett. 2010;4(4):195‐201. 20583968 10.2174/187231210792928251 PMC4486004

[42] Shen DW , Pouliot LM , Hall MD , Gottesman MM . Cisplatin resistance: a cellular self‐defense mechanism resulting from multiple epigenetic and genetic changes. Pharmacol Rev. 2012;64(3):706‐721. 22659329 10.1124/pr.111.005637 PMC3400836

[43] Lei ZN , Teng QX , Zhang W , et al. Establishment and characterization of a topotecan resistant non‐small cell lung cancer NCI‐H460/TPT10 cell line. Front Cell Dev Biol. 2020;8:607275. 33425914 10.3389/fcell.2020.607275 PMC7786180

[44] Wu ZX , Teng QX , Cai CY , et al. Tepotinib reverses ABCB1‐mediated multidrug resistance in cancer cells. Biochem Pharmacol. 2019;166:120‐127. 31078601 10.1016/j.bcp.2019.05.015

