# Supplementary material for: Overcoming multidrug resistance by knockout of ABCB1 gene using CRISPR/Cas9 system in SW620/Ad300 colorectal cancer cells
Source: MedComm (2020). 2021 Dec 16;2(4):765–77. doi: 10.1002/mco2.106 (PMC8706751; doi:10.1002/mco2.106)
Supplement: Supplementary file 1 — SUPPORTING INFORMATION [file MCO2-2-765-s001.docx]

**Overcoming Multidrug Resistance by Knockout of ABCB1 Gene Using CRISPR/Cas9 System in SW620/Ad300 Colorectal Cancer Cells**

Zi-Ning Lei^1^, Qiu-Xu Teng^1^, Zhuo-Xun Wu^1^, Feng-Feng Ping^2^, Peng Song^3^, John N.D. Wurpel^1,*^, and Zhe-Sheng Chen^1,*^

^1^ Department of Pharmaceutical Sciences, College of Pharmacy and Health Sciences, St. John’s University, Queens, NY 11439, USA.

^2^ Department of Reproductive Medicine, Wuxi People’s Hospital Affiliated to Nanjing Medical University, Wu-xi, Jiangsu 214023, P.R. China.

^3^Key Laboratory of Prevention and Treatment for Chronic Diseases by TCM in Gansu Province, Affiliated Hospital of Gansu University of Chinese Medicine, Lanzhou 730000, P.R. China

**Corresponding authors:**

- Dr. Zhe-Sheng Chen, M.D., Ph.D., Department of Pharmaceutical Sciences, St. John’s University, 8000 Utopia Parkway, Queens, New York, NY 11439, USA. Tel: 1-718-990-1432; Fax: 1-718-990-1877; E-mail: [chenz@stjohns.edu](mailto:chenz@stjohns.edu)
- Dr. John N.D. Wurpel, Ph.D., Department of Pharmaceutical Sciences, St. John’s University, 8000 Utopia Parkway, Queens, New York, NY 11439, USA. Tel: 1-718-990-5265; Fax: 1-718-990-1877; E-mail: [wurpelj@stjohns.edu](mailto:wurpelj@stjohns.edu)

**Supplementary Materials**

**
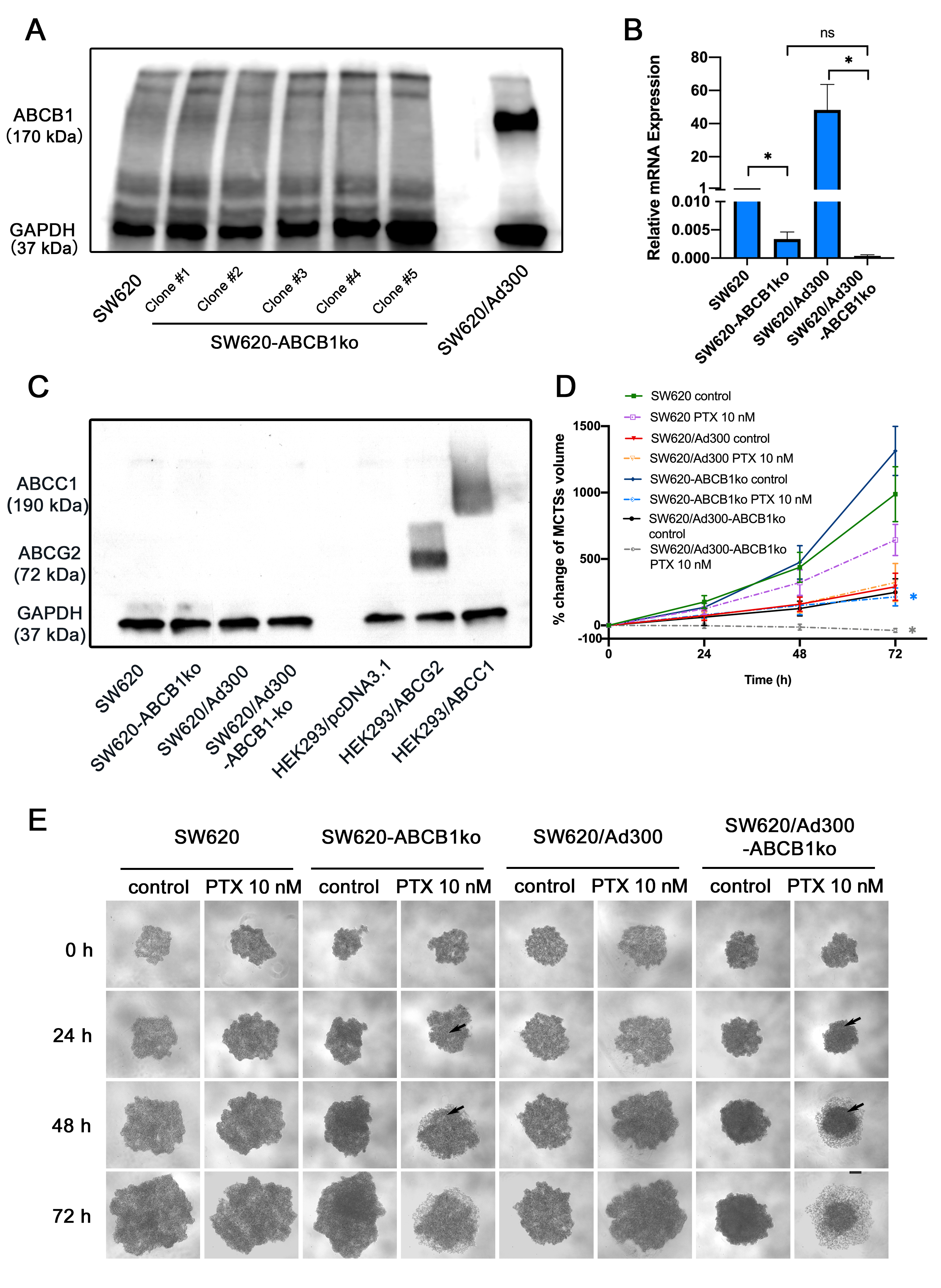
**

**Figure S1 ABCB1 Gene Knockout in SW620 Cells. (A)** Western blot on the expression levels of ABCB1 in SW620, SW620/Ad300, and five different clones obtained from SW620 cells with ABCB1 gene knockout by CRISPR/Cas9 system. GAPDH was used as a loading control. Clone #5 was selected as stably *ABCB1* gene knockout subline of SW620, termed SW620-ABCB1ko, and was used for further mRNA expression and drug sensitivity analysis. **(B)** mRNA expression levels measured by RT-qPCR were normalized by the expression of GAPDH. Relative mRNA expression was presented as fold change versus SW620. * indicates p < 0.05. **(C)** Western blot on the expression levels of ABCG2 and ABCC1 in SW620, SW620/AD300, SW620-ABCB1ko and SW620/Ad300-ABCB1ko cells. HEK293/pcDNA3.1 was used as a negative control with no ABCG2 or ABCC1 expression. HEK293/ABCG2 and HEK293/ABCC1 were used as positive controls for ABCG2 and ABCC1 expressions, respectively. **(D)** Change of MCTSs volumes after treatment with either vehicle control (culture media) or 10 nM paclitaxel at time points 0, 24, 48, and 72 h. Percentage of MCTSs volume was calculated by (spheroid volume – spheroid volume at timepoint-0)/spheroid volume at timepoint-0 × 100%. Data points and error bars represented the mean and standard deviation obtained from two independent experiments performed with 6 replicates. The * labels were shown in the corresponding color to the figure legends. * indicates p < 0.05 compared to the control group of the corresponding cell line. (E) Representative images of the MCTSs in the control group and 10 nM paclitaxel-treated group. The loosely associated dead cells at the outer layer of spheroids were pointed by the black arrows. Scale bar represented 200 µm.


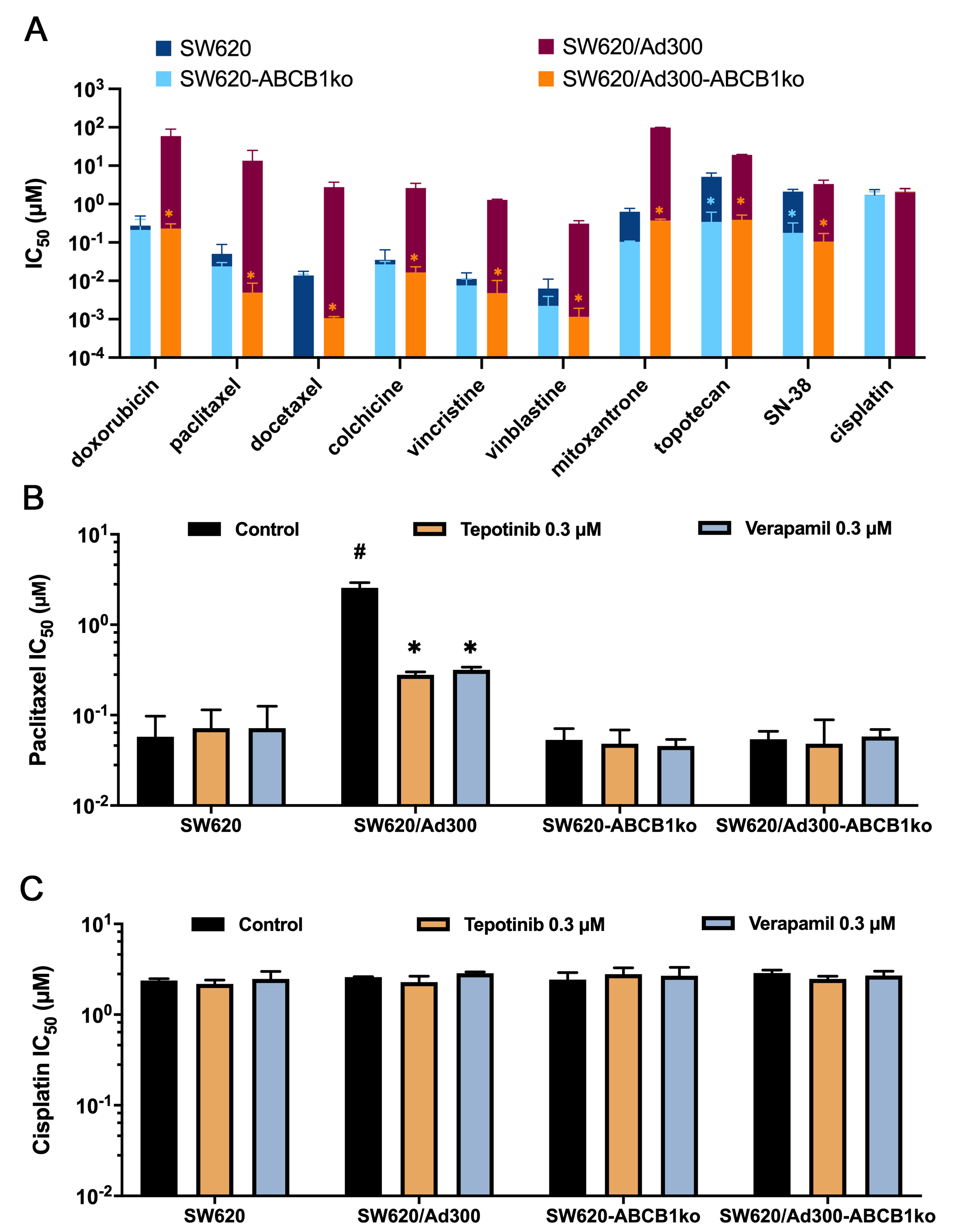


**Figure S2 The drug resistance spectrum and reversal tests in *ABCB1* gene knockout cells.** **(A)** The IC_50_ values of anticancer drugs were obtained from MTT assay against SW620, SW620-ABCB1ko, SW620/Ad300, and SW620/Ad300-ABCB1ko cells. The data columns of SW620-ABCB1ko were superimposed with the corresponding ones of SW620 cells, and the data columns of SW620/Ad300-ABCB1ko were superimposed with the corresponding ones of SW620/Ad300 cells for comparison. The columns and error bars represented the mean and SD of three independent experiments in triplicate. * p < 0.05 comparing the *ABCB1* knockout subline with the corresponding cell line without *ABCB1* knockout. **(B) (C)** Change of cell viability determined by MTT assay in response to different concentrations of paclitaxel **(B)** and cisplatin **(C)**, with or without the presence of tepotinib or verapamil. ^#^ indicates p < 0.05 compared to the control group of SW620 cells. * indicates p < 0.05 comparing the groups with tepotinib to the control group of the corresponding cell line.
